# Design and Optimization of Composite Thermal Insulation Layers for Mine Ecological Restoration Under Simulated Solar Radiation: Integrating Response Surface Screening with Gaussian Process Bayesian Optimization

**DOI:** 10.3390/ma19163388

**Published:** 2026-08-10

**Authors:** Ziqiang Zhou, Xuemei Jia, Guoxin Zhang, Tao Wen, Li Ma, Yun Guo, Jing Ge

**Affiliations:** 1 Geological Hazards Prevention Institute, Gansu Academy of Sciences, Lanzhou 730000, China; zzq01118@163.com (Z.Z.); guoxinzh@163.com (G.Z.); 15294150089@163.com (L.M.); xnguoyun@163.com (Y.G.); gejing@lzu.edu.cn (J.G.); 2 College of Earth and Environmental Sciences, Lanzhou University, No. 222, Tianshui South Road, Lanzhou 730000, China; 3 School of Civil and Architectural Engineering, Yangtze Normal University, Chongqing 408100, China

**Keywords:** mine ecological restoration, thermal insulation layer, two-stage optimization, thermal regulation, solar radiation simulation, sustainable materials

## Abstract

Thermal regulation of surface soil is critical for vegetation establishment in degraded mining environments, yet the systematic design of insulation layers tailored to mine restoration remains underdeveloped. Fifteen candidate thermal insulation materials from six categories were evaluated under simulated solar radiation, and a two-stage optimization framework was established. The first stage employed response surface methodology (RSM) for preliminary screening; the second used Gaussian process regression with Bayesian optimization (GPR-BO) for mixture refinement. RSM identified hollow glass microspheres as the strongest positive contributor and wood chips as the most detrimental component. The GPR-BO framework yielded an optimal formulation achieving *T*_90_ = 12.80 °C, heating rate *v* = 0.0311 °C/min, and heat resistance efficiency *η* = 65.85%, with R^2^ exceeding 0.95 for all response variables. The observed thermal regulation arose from the synergy of three mechanisms: surface radiative heat suppression, internal conductive path interruption, and transient thermal buffering. These findings offer a practical design route for high-performance insulation layers in cold-region mine ecological restoration.

## 1. Introduction

Mining activities provide essential resources but also cause topsoil stripping, surface exposure, soil compaction, and shallow-water deficit, all of which severely impede vegetation establishment. These impacts are exacerbated in high-altitude, cold-arid, high-radiation mining areas, where reconstructed soils are thermally unstable and prone to rapid daytime heating and evaporation. Under such conditions, the shallow restoration layer experiences strong thermal fluctuations that further restrict seed germination, seedling survival, and root penetration. Recent work has emphasized that mine ecological restoration is shifting from simple revegetation toward integrated soil reconstruction and microenvironment regulation, particularly in climatically harsh mining regions [1,2]. In cold-region reclamation, insulation covers play a critical role in controlling the thermal regime of the reclaimed system [3]. Accordingly, material design for ecological restoration covers in cold mining areas must simultaneously reduce short-term heat input, delay downward heat transfer, and mitigate shallow-layer water loss [1,4].

The thermal behavior of a compacted mine restoration cover can be interpreted through three coupled physical processes. First, the surface radiative balance controls the solar energy entering the system: higher surface reflectance directly reduces absorbed heat flux, which is why optical components such as hollow glass microspheres and TiO_2_ can suppress surface heating. Second, heat propagates downward mainly through conduction, governed by effective thermal conductivity (reduced by air-filled pores that extend conductive pathways) and volumetric heat capacity (which delays the thermal response). Third, phase change materials provide transient thermal storage by absorbing latent heat at the phase-transition temperature, buffering transient thermal peaks. These three processes correspond to three distinct material-design targets: maximizing reflectance (optical function), minimizing conductivity (porous-skeleton function), and enhancing heat capacity (thermal-buffering function). Effective insulation therefore requires the simultaneous engineering of all three functions rather than optimization of a single parameter.

In mine reclamation, these targets are further constrained by compaction, particle gradation, pore evolution, wind and rainfall erosion, and compatibility with in situ soils. Reviews on mine Technosols have highlighted that restoration substrates must combine erosion resistance, water retention, anti-desiccation capacity, and structural stability [4], while field studies on reclaimed open-pit coal mines have confirmed that different reclamation modes substantially alter soil physical properties and infiltration behavior [5]. Accordingly, the design of insulation-layer materials for mine restoration should integrate thermal regulation, hydrothermal buffering, structural support, and engineering feasibility, rather than simply transplanting conventional building-insulation concepts [4,5,6].

Among candidate material sources, solid wastes have attracted considerable attention due to their low cost, large availability, and potential for resource recycling. Recent reviews have shown that fly ash, coal gangue, tailings, phosphogypsum, and ash–slag residues can be converted into thermal insulation materials via foaming, sintering, or lightweight aggregate routes. These processes mainly improve thermal performance by increasing closed porosity, reducing density, and extending heat-transfer paths [7]. For example, coal-gangue-based foamed ceramics with a thermal conductivity of approximately 0.197 W/(m·K) have been reported [8]. In addition, CaO–Al_2_O_3_–SiO_2_-based porous ceramics co-utilizing phosphogypsum and coal gangue have also been successfully demonstrated [9]. More broadly, solid-waste-derived porous ceramics have been extended to applications in thermal insulation, sound absorption, and vertical planting [10,11]. However, most of these studies focus on building material applications. In such cases, thermal performance is typically evaluated on sintered or foamed products under uncompressed conditions. Their behavior under compacted, soil-coupled, and moisture-affected conditions, as encountered in mine restoration covers, remains largely unexplored. Furthermore, the high-temperature sintering or chemical foaming processes used in these studies are difficult to directly apply in field restoration scenarios. In practice, materials are usually placed as granular mixtures and compacted in situ. Therefore, whether the favorable thermal properties of solid-waste-derived materials can be maintained in a compacted, multi-component restoration layer remains an open question.

Porous materials constitute another major route for insulation-layer construction. They rely on the low thermal conductivity of trapped air and the tortuous heat-transfer paths created by multiscale pore networks [12]. Industrial and mining wastes such as fly ash and tailings have been successfully converted into structurally stable porous thermal barriers via foam-gelcasting and gel-casting routes [13,14]. This suggests their potential as the primary skeleton of the insulation layer. A critical concern is that pore structures responsible for low thermal conductivity are typically characterized on uncompacted or sintered specimens. Under the compaction required for restoration-layer placement, partial pore collapse and increased inter-particle contact may significantly reduce the insulation performance. However, this compaction sensitivity has rarely been quantified.

Optical materials regulate thermal behavior mainly by reducing radiative heat gain. Hollow glass microspheres, hollow TiO_2_ microspheres, and TiO_2_/SiO_2_ core–shell particles can reduce effective thermal conductivity through enclosed cavities while increasing solar reflectance via engineered surface structures [15,16,17,18,19]. These results indicate that optical components are promising for mitigating surface heating. However, most reported reflectance and thermal-barrier performances were measured on clean, laboratory-prepared surfaces. In mine restoration covers, dust accumulation, moisture variation, and irregular compaction geometry may degrade radiative performance. The long-term stability of optical functionality under field weathering conditions remains insufficiently studied.

Bio-based materials offer low density, renewability, and potential hygrothermal buffering through plant-cell lumens and fiber-interspace pores [20,21]. Multifunctional systems combining corncob, geopolymer, and silica aerogel [22], straw–aerogel composites [23], and cellulose-based aerogel fibers [24,25] illustrate the diversity of this design space. However, two key challenges limit their direct application in mine restoration. First, thermal performance is strongly moisture-dependent, and dry-state conductivity may underestimate field heat transfer under realistic moisture conditions. Second, long-term durability in soil-contact environments remains unclear, where microbial degradation, wet–dry cycling, and compaction-induced damage may co-occur.

Phase change materials (PCMs) provide a complementary thermal-regulation mechanism by storing and releasing latent heat to mitigate transient thermal fluctuations [26,27]. In mine ecological restoration, PCMs can function as thermal-buffering components. However, their effectiveness is strongly constrained by the match between phase-transition temperature and local ground-temperature conditions. Many PCMs developed for building applications have transition temperatures in the 20–30 °C range. In contrast, near-surface soil temperatures in cold-region mines may rarely reach these levels. When the phase-transition temperature is not reached during diurnal cycles, the latent-heat mechanism remains inactive, and PCMs provide only sensible-heat buffering, offering limited advantage over conventional fillers. This temperature-matching issue has not been sufficiently addressed in existing PCM insulation studies.

Collectively, the four material categories reviewed above offer distinct and potentially complementary thermal-regulation mechanisms. However, a consistent limitation across the literature is that performance has been evaluated almost exclusively on single-category specimens under conditions substantially different from compacted, multi-component mine restoration layers. The extent to which these mechanisms persist, degrade, or interact when materials are combined, compacted, and coupled with in situ soil remains an open question.

Beyond material selection, a further challenge lies in designing rational material combinations. Traditional trial-and-error formulation methods are inefficient for multi-component systems in which skeleton materials, secondary skeletons, and fillers interact nonlinearly. Response surface methodology (RSM) has been widely applied in insulation mortars, foamed gypsum, foam ceramics, and solid-waste foam concretes because it can identify significant factors, fit quadratic response surfaces, and perform multi-objective optimization with relatively few experiments [28,29,30,31]. These studies demonstrate the effectiveness of statistical experimental design for small-sample, multi-factor systems, but also reveal the limitations of predefined polynomial models when the formulation space becomes highly nonlinear.

To overcome these limitations, machine-learning-based material design has devel-oped rapidly in recent years. Compared with RSM, ML models are not restricted to a fixed functional form and are better suited to capturing complex nonlinear mappings. Logbi et al. compared RSM and ANN for eco-friendly insulating bricks and showed the ad-vantages of intelligent models for coupled thermo-mechanical prediction [32]. Noui et al. combined central composite design with neural networks to optimize the thermal and mechanical behavior of multi-waste foam geopolymers [33]. More directly in the field of thermal insulation, Mukherjee et al. developed a supervised ML framework to predict the effective thermal conductivity of hollow-glass-microsphere composites [34]. Rahman et al. compared five ML methods for low-temperature multilayer insulation and found that SVR performed best when material type, cold-side vacuum pressure, and layer density were used as descriptors [35]. and Fellah et al. reported that RF and XGBoost could reach pre-diction accuracies above 94% in thermal-insulation-based energy and comfort assessment [36]. More broadly, Zhu et al. showed that machine learning is accelerating the inverse de-sign of thermal metamaterials and heat-management systems [37]. Among these approaches, Gaussian process regression (GPR) and Bayesian optimization (BO) are especially suitable for mine-restoration insulation-layer design, where experiments are expensive, datasets are small, and objectives are multiple. GPR provides both predictive means and uncertainty estimates, whereas BO uses acquisition functions to balance exploration of uncertain regions and exploitation of high-performing regions. Kiyohara et al. developed a GP-based Bayesian optimization framework assisted by deep learning for material design [38], and Alvi et al. further proposed hierarchical GP-BO and deep-GP cost-aware BO schemes for complex materials campaigns [39,40]. For mine ecological restoration insulation layers composed of multiple interacting materials, such a strategy enables data-efficient screening, surrogate modeling, and targeted optimization under uncertainty.

Despite these advances, most existing studies still focus on building envelopes, reflective coatings, foam ceramics, or single insulation boards. Systematic research tailored to mine ecological restoration cover systems remains limited. In mines, restoration insulation layers are usually compacted, overlain by soil, and coupled with an in situ substrate. These conditions create a heat-transfer environment that differs substantially from conventional construction applications. In addition, the design of mine restoration materials must consider several requirements simultaneously, including thermal performance, waste valorization, structural compatibility, ecological safety, and field constructability. Against this background, the present study was designed to test three hypotheses.

The first is the multi-mechanism synergy hypothesis: the thermal insulation performance of a compacted mine restoration layer results from the synergy of surface radiative heat suppression, internal conductive path interruption, and transient thermal buffering, rather than from any single material property. If this hypothesis is valid, no single material category should achieve optimal overall performance, and the best-performing formulations should contain components addressing all three mechanisms. The second is the differential material contribution hypothesis: optical components, especially hollow glass microspheres, are expected to contribute most strongly to overall performance owing to their combined reflectance and low-conductivity effects, whereas certain bio-based materials with high thermal diffusivity may negatively affect performance under compaction. If valid, RSM screening should rank combinations containing optical components highest. The third is the two-stage optimization efficiency hypothesis: a sequential framework combining RSM-based statistical screening with GPR-BO-driven mixture optimization should identify near-optimal formulations with fewer experimental iterations than exhaustive combinatorial testing or trial-and-error approaches. If valid, the GPR-BO surrogate model should achieve high predictive accuracy and converge to a Pareto-optimal formulation within a limited number of BO iterations.

To test these hypotheses, a candidate material system was established comprising 15 materials from six categories: soil-derived materials, solid wastes, biomass materials, porous materials, optical materials, and phase change materials. Indoor lighting simulation tests were conducted under simulated solar radiation. A two-stage strategy integrating response surface analysis, multi-response desirability evaluation, and GPR-BO was adopted, enabling preliminary screening, key-factor identification, and formulation optimization. By testing explicit and falsifiable hypotheses rather than relying solely on exploratory comparisons, this study aims to identify an optimized formulation, clarify the underlying design logic, and evaluate whether the proposed multi-mechanism framework provides a coherent basis for the rational design of thermal insulation layers in mine ecological restoration.

## 2. Materials and Test Plans

### 2.1. Materials

#### 2.1.1. Soil

**Figure 1:**
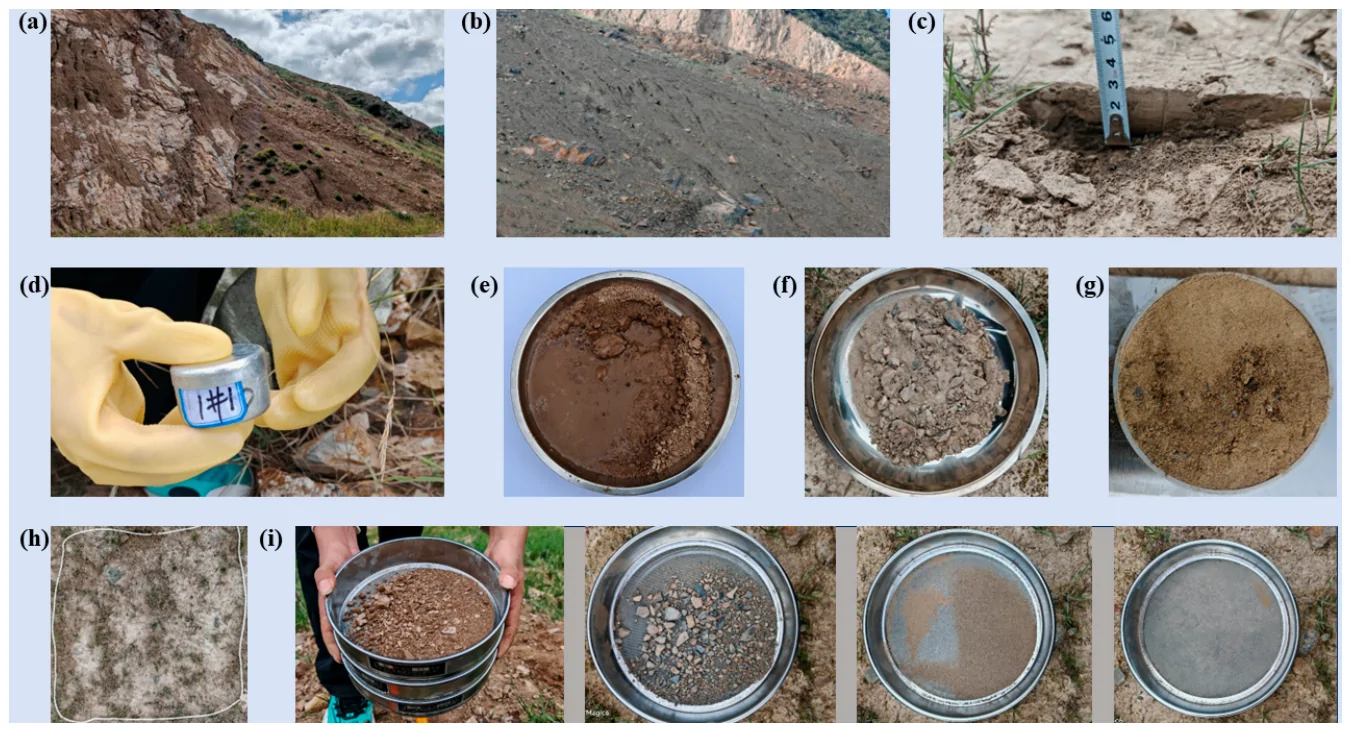
Soil sampling and field testing in mining areas: (**a**) Soil sampling sites; (**b**) Mine surface; (**c**) Subsurface moisture conditions; (**d**) Soil sample; (**e**) Moisture content test—before burning; (**f**) Moisture content test—after burning; (**g**) Ring knife method; (**h**) Vegetation growth status; (**i**) Particle size distribution.

The soil samples were collected from a high-altitude arid region of the Yellow River Basin in Gansu Province, China (Figure 1a). The study area has a highland continental monsoon climate characterized by high elevation, low temperature, and a long winter with a short summer. Long-term meteorological records indicate a mean annual temperature of 5.1 °C (extreme maximum 33.5 °C, extreme minimum −23.4 °C), mean annual precipitation of 400 mm, mean annual evaporation of 1221.9 mm, annual sunshine duration of 2372.8 h, average relative humidity of 65%, and maximum wind speed of 24 m/s.

**Figure 2:**
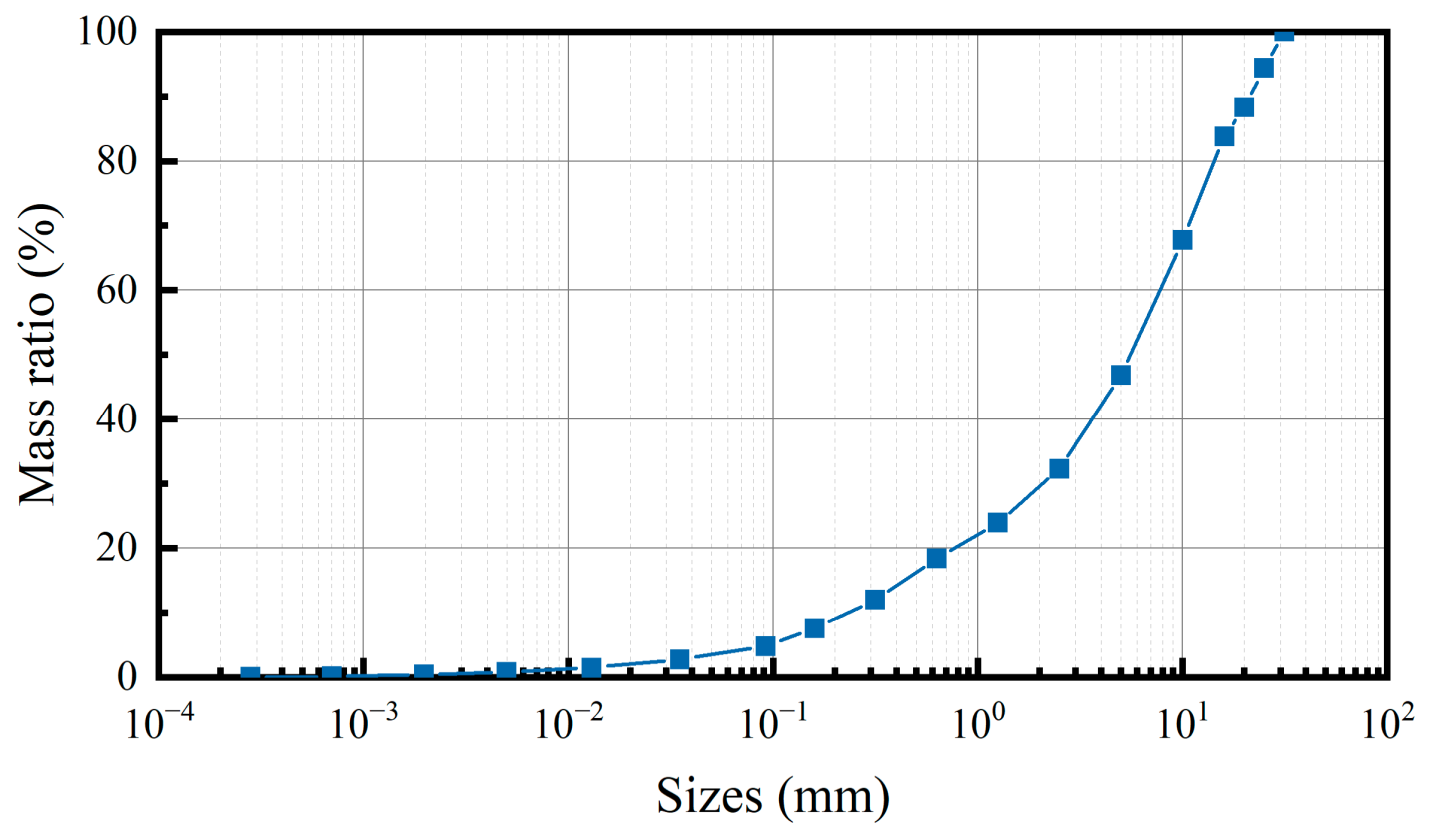
The gradation curve for the soil sample.

Mining activities in the study area resulted in severe topsoil loss, which inhibited vegetation establishment and markedly damaged the local ecological environment and soil and water conservation capacity (Figure 1b). In addition, the soil layer at a depth of 3 cm below the surface was found to be dehydrated and compacted (Figure 1c). Soil samples were collected within the mining area, and in situ tests of soil properties were carried out (Figure 1d). Soil moisture content was determined in the field using the combustion method (Figure 1e,f). Undisturbed soil samples were collected using the ring knife method, sealed immediately after sampling, and transported to the laboratory for density determination (Figure 1g). Soil moisture, density, and compaction characteristics were determined in accordance with GB/T 50123-2019 [41]. Vegetation growth status in the mining area was investigated using a 1 m × 1 m quadrat (Figure 1h). The particle size distribution of the soil samples was determined by sieving (Figure 1i); analysis of finer soil particles was completed in the laboratory. The soil sample has a density of 2.052 g/cm^3^, water content of 9.0%, and plant growth status of 12 plants/m^2^. The gradation curve for the soil sample is shown in Figure 2.

#### 2.1.2. Thermal Insulation Material

**Figure 3:**
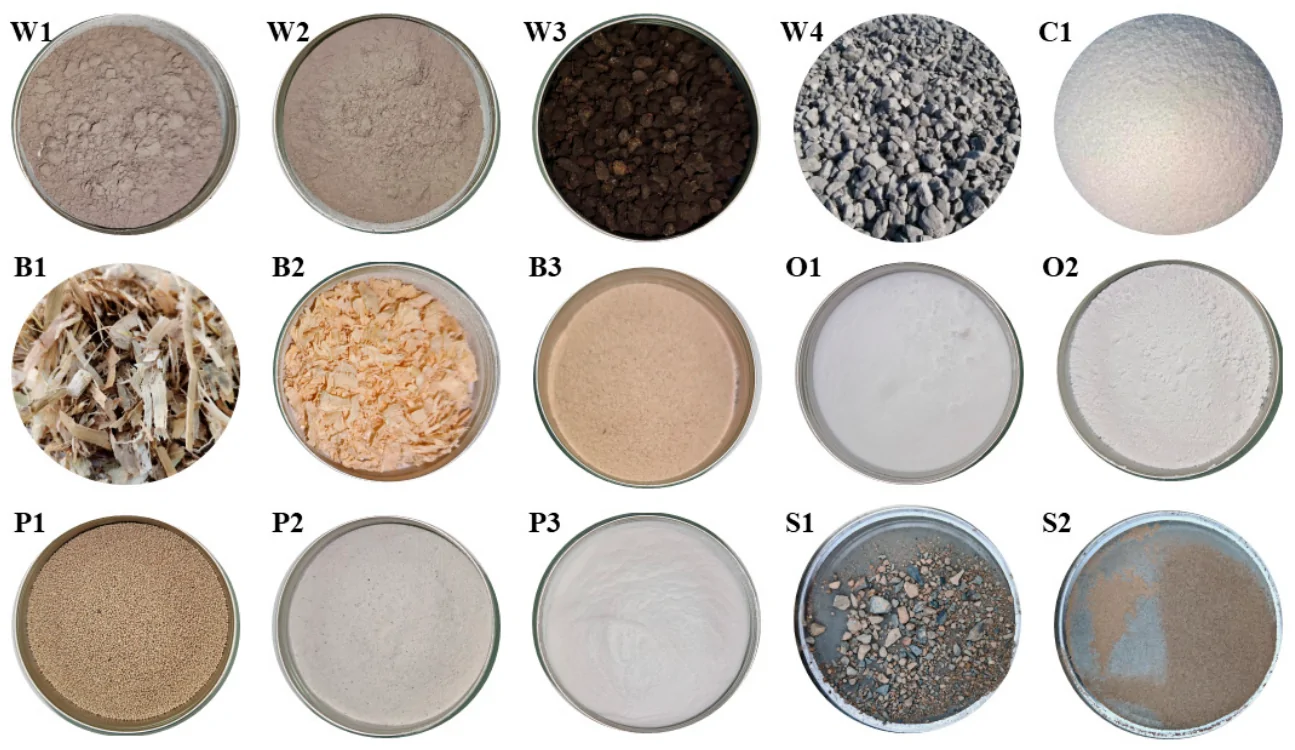
Thermal insulation materials.

A total of 15 thermal insulation materials were used, grouped into six categories: soil samples, solid waste materials, biomass materials, porous materials, optical materials, and phase change materials. Based on sieve size, the untreated soil samples were divided into soil particles ≤ 5 mm (S1) and tailings > 5 mm (S2). The solid waste materials included fly ash (W1), phosphogypsum (W2), coal slag (W3), and coal gangue (W4). The biomass materials included straw (B1), wood chips (B2), and recycled wood flour (B3). The porous materials included expanded clay aggregates (P1), diatomaceous earth (P2), and aerogel (P3). The optical materials included hollow glass microspheres (O1) and titanium dioxide (O2). Phase change paraffin (C1) was used as the phase change material; above its melting point, liquid paraffin fills inter-particle voids and suppresses convective heat transfer within pores, providing a modest thermal-resistance contribution even when latent heat storage is inactive [42]. The materials are shown in Figure 3, and their technical properties are listed in Table 1. Thermal conductivity was measured in accordance with GB/T 10297-2015 [43].

The properties listed in Table 1 were selected because they directly govern heat transport in compacted granular layers. Bulk density controls the solid-phase volume fraction and the inter-particle contact area. Thermal conductivity determines the intrinsic conductive resistance of each component. Porosity reflects the capacity for air occlusion, which disrupts conductive heat-transfer pathways.

### 2.2. Test Methods

#### 2.2.1. Method of Specimen Preparation

Insulation layer specimens (ILSs) were prepared by compaction testing to simulate the in situ compaction state of the ecological restoration layer, in accordance with GB/T 50123-2019 [41].

**Figure 4:**
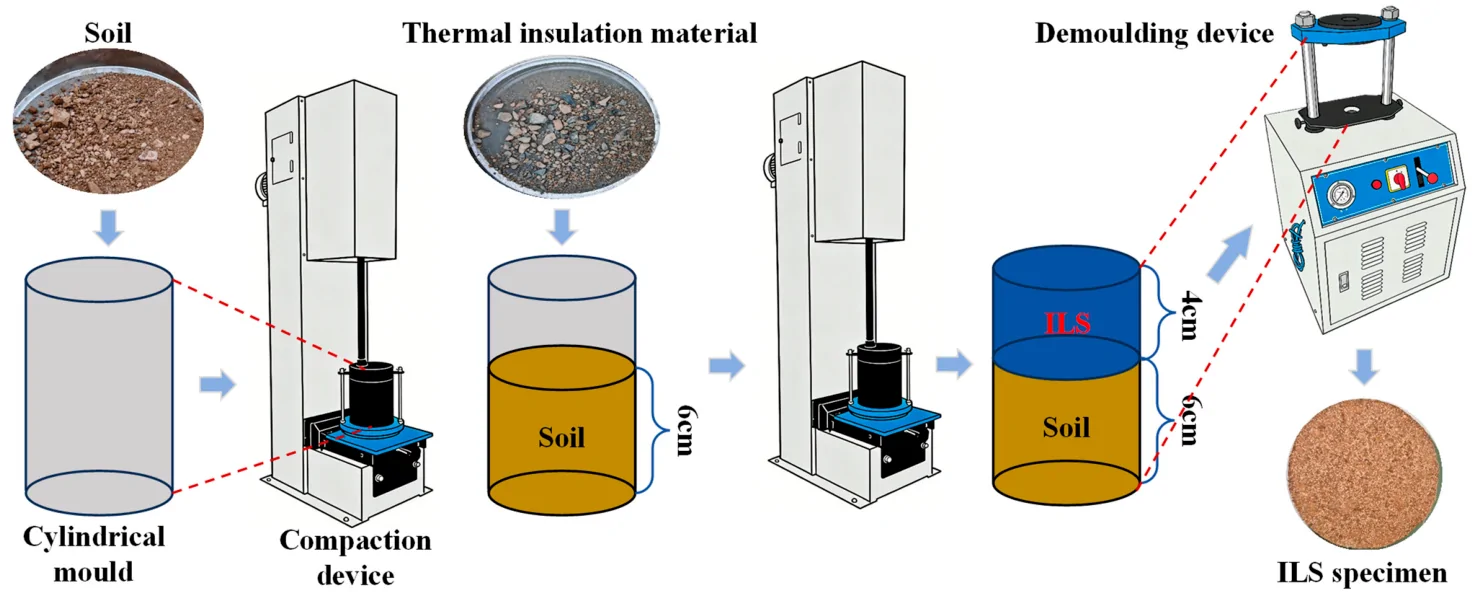
The molding process for ILSs.

Materials were dried at 105 °C to constant mass. Undisturbed soil was placed into a cylindrical mold (inner diameter 100 mm, height 140 mm) and compacted to a depth of 6 cm. Insulation layer materials were prepared according to the designed volume ratio, mixed with water to achieve optimum moisture content, placed into the mold, and compacted to a depth of 4 cm. The specimen was then demolded (Figure 4). Three replicate specimens were prepared for each material combination, and all indices represent mean values.

#### 2.2.2. Indoor Lighting Simulation Tests

**Figure 5:**
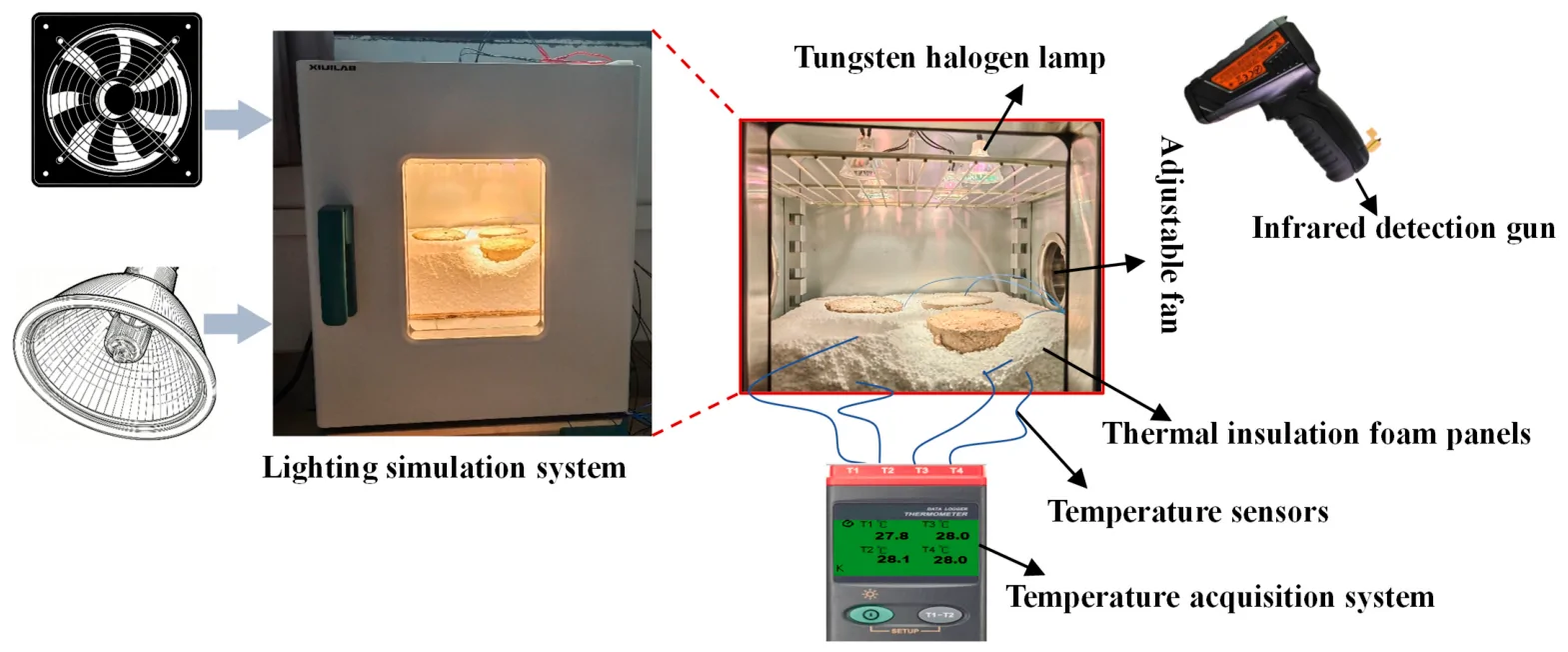
Indoor lighting simulation test equipment.

To reproduce the ecological conditions of a mining area, an indoor lighting simulation test was designed and conducted using a self-developed lighting simulation system (Figure 5). Since no standardized protocol exists for solar simulation testing of compacted insulation layers, the experimental setup followed the approach proposed by Yi et al. [44], who used a tungsten halogen lamp to simulate solar radiation under controlled laboratory conditions. In the present study, a tungsten halogen lamp was employed as the solar radiation source. An adjustable fan was used to control wind speed. In addition, a constant-temperature oven system was used to simulate ground temperature conditions.

An indoor lighting simulation test was conducted using a self-developed lighting simulation system (Figure 5). Since no standardized protocol exists for solar simulation testing of compacted insulation layers, the experimental setup followed the approach of Yi et al. [44], who used a tungsten halogen lamp to simulate solar radiation under controlled laboratory conditions. The lamp (rated power 1000 W, luminous flux 18,000 lm) produces a continuous emission spectrum from ultraviolet to near-infrared wavelengths. Although its spectrum is shifted toward the infrared compared with the natural solar spectrum, it provides reasonable coverage of the near-infrared band, which is closely associated with radiative surface heating. The irradiance at the specimen surface was approximately 200 W/m^2^, close to the annual mean solar irradiance of the study area (206.73 W/m^2^). The vertical distance between the lamp and specimen surface was 20 cm. Thermal insulation foam panels were arranged around the specimen to reduce lateral heat transfer, enabling the heat transfer process to approximate one-dimensional vertical conduction. Internal specimen temperatures were measured using type K thermocouples (model TP K01, range −50 to 200 °C, accuracy ±1 °C) embedded at the required depths and connected to a multichannel data logger. Surface temperature was measured using a Delixi infrared thermometer (accuracy ±0.1 °C, Yueqing, Zhejiang, China). An adjustable fan controlled wind speed, and a constant-temperature oven system simulated ground temperature conditions.

The wind speed generated by the fan was set to 1 m/s, which was close to the annual average wind speed in the study area. The oven temperature was set at 10 °C to approximate the average maximum air temperature. After the indoor illumination simulation test was initiated, the surface temperature of the ILS was measured using the infrared thermometer. Internal temperatures were recorded by temperature sensors embedded at depths of 4 cm and 10 cm, corresponding to the bottom of the insulation layer and the bottom of the soil layer, respectively.

### 2.3. Test Plan

#### 2.3.1. Research Framework

**Figure 6:**
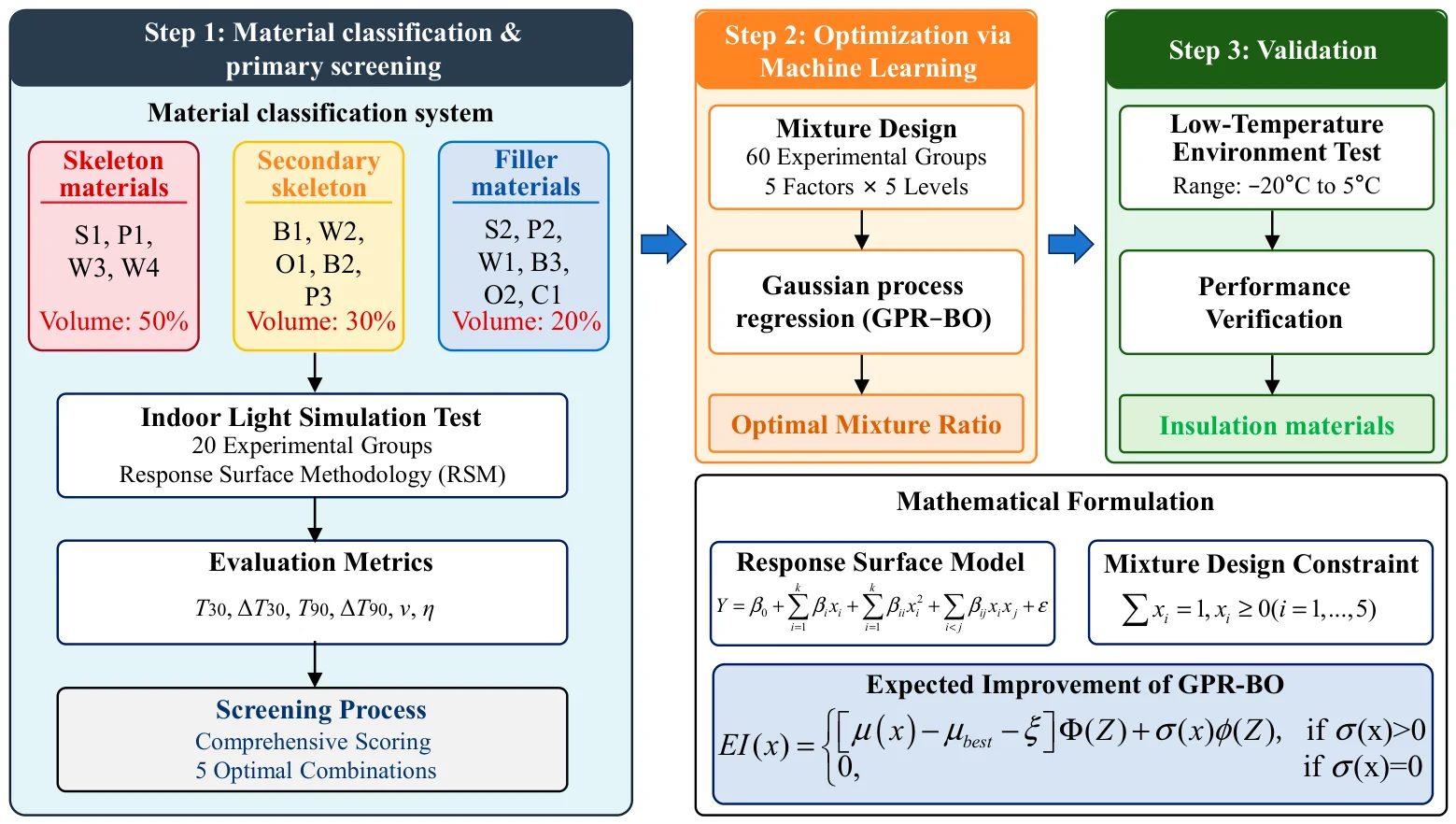
Framework for optimizing thermal insulation materials.

To establish an efficient design strategy for the ecological restoration insulation layer, the research program was divided into two successive stages: preliminary material screening and material proportion optimization. Candidate materials were first classified according to their functional roles within the insulation layer. S1, P1, W3, and W4 were defined as primary skeleton materials, mainly providing structural support and framework. B1, W2, O1, B2, and P3 were regarded as secondary skeleton materials, expected to improve pore structure and thermal resistance. S2, W1, P2, B3, O2, and C1 were used as filling materials to regulate compactness, radiative properties, and thermal storage behavior. The research framework is shown in Figure 6.

In the preliminary screening stage, the volumetric ratio of primary skeleton, secondary skeleton, and filling material was fixed at 50%, 30%, and 20%, respectively. Under this condition, 20 groups of indoor illumination simulation tests (Table 2) were conducted. The thermal insulation performance of each specimen was evaluated using six indices: temperature after 30 min of illumination (*T*_30_), temperature difference after 30 min (Δ*T*_30_), temperature after 90 min of illumination (*T*_90_), temperature difference after 90 min (Δ*T*_90_), average heating rate (*v*), and heat resistance efficiency (*η*). Here, Δ*T*(*t*) = *T*(*t*) − *T*(0), *v* = [*T*(*t*) − *T*(0)]/*t*, and η is defined in Equation (1). Based on response surface analysis and multi-response comprehensive evaluation, the material combinations with superior overall performance were identified for the next stage.

(1) $$\eta =\frac{T_{b}(t)-T_{s}(t)}{T_{b}(t)-T_{air}}\times 100\%$$

where *T*(*t*) is the temperature of the test specimen after *t* minutes of illumination, *T*_b_(*t*) is the temperature of the control specimen after *t* minutes of illumination, *T*_s_(*t*) is the temperature of the ILS after *t* minutes of illumination, and *T*_air_ is the ambient temperature.

A Gaussian Process Regression with Bayesian Optimization (GPR-BO) framework was employed to systematically optimize thermal insulation material formulations. An initial dataset of 32 samples was generated using a three-factor design. GPR was adopted as the surrogate model to describe the nonlinear relationships between formulation parameters and material performance. A composite kernel consisting of radial basis function and white noise components captured both smooth underlying trends and stochastic noise. Prior to model training, all input variables were standardized, and model robustness was ensured through 5-fold cross-validation with multiple optimizer restarts to avoid local optima. Four response variables were considered: *T*_90_, *v*, *η*, and cost (*C*). *T*_90_, *v*, and *C* were treated as minimization objectives, whereas η was treated as a maximization objective, forming a multi-objective optimization problem. The Expected Improvement acquisition function guided sample selection by balancing exploration and exploitation. Fifteen iterative evaluations were performed to progressively refine the surrogate model. Finally, Pareto front analysis was conducted to characterize trade-offs among competing objectives, and the optimal formulation was identified as a compromise solution achieving balanced overall performance.

#### 2.3.2. Preliminary Screening of Insulation Materials

In the preliminary screening stage, response surface analysis was employed to establish the relationship between material combination and thermal response. Since the tested system involved several interacting factors, response surface methodology was used to quantify the main effects, interaction effects, and nonlinear trends of the selected variables, as expressed in Equation (2). The model coefficients were estimated using the least squares method, and model fit was evaluated using the coefficient of determination, R^2^.

(2) $$Y=\beta_{0}+\sum_{i=1}^{k}\beta_{i}x_{i}+\sum_{i=1}^{k}\beta_{ii}x_{i}^{2}+\sum_{i=1}^{k}\sum_{j=i+1}^{k}\beta_{ij}x_{i}x_{j}+\varepsilon$$

where *Y* is the predicted response, *x*_i_ and *x*_j_ are the coded independent variables, *β*_0_ is the intercept term, *β*_i_ is the linear coefficient, *β*_ii_ is the quadratic coefficient, *β*_ij_ is the interaction coefficient, and *ε* is the random error.

The desirability function approach was employed to calculate comprehensive performance scores. This methodology transforms each response variable into a dimensionless desirability value (*d*_i_) between 0 and 1, where *d*_i_ = 1 represents the ideal value and *d*_i_ = 0 represents the worst acceptable value. The overall desirability (*D*) was calculated as the weighted geometric mean of individual desirability values. Weights were assigned according to indicator relevance to insulation-layer design: *T*_90_, *v*, and *η* were each assigned 20% (representing steady-state thermal resistance, transient heating rate, and overall heat resistance efficiency); *T*_30_ and Δ*T*_90_ were each assigned 15% as secondary indicators; and Δ*T*_30_ was assigned 10% because the 30 min response is more affected by surface radiation and is less representative of through-thickness insulation. These weights were used only for preliminary RSM screening; the subsequent GPR-BO stage employed Pareto-based multi-objective optimization with each response treated independently.

#### 2.3.3. Mixture Optimization Based on Machine Learning

In the optimization stage, a GPR model was established to describe the relationship between the composition of the mixture and its overall thermal insulation performance. The volume ratios of the primary skeleton, secondary skeleton and filler were treated as three independent formulation variables, *x*_1_, *x*_2_ and *x*_3_, with ranges of 0–0.25, 0–0.10 and 0–0.25 respectively. The base soil (S1, soil from the in situ mining area) constitutes the remainder of the mixture, defined as 1–*x*_1_–*x*_2_–*x*_3_, and is not treated as an independent variable. The total composition is constrained to 100%. The overall desirability index *D*, calculated from the six thermal response indices, was adopted as the target output. In this way, the multi-objective optimization problem was transformed into a single comprehensive response optimization problem.

The cost objective, C (yuan/t), was calculated as the sum of the unit price of each component material multiplied by its dosage in the insulation-layer mixture. The unit prices were obtained from online market surveys conducted in the second quarter of 2026 using the Alibaba 1688 platform (http://www.1688.com) and Baidu Ai Procurement (https://aicaigou.baidu.com). The base soil (S1/S2) was assumed to be available on site at zero material cost. Transportation costs were excluded because material sourcing distance is site-dependent.

GPR is a nonparametric Bayesian learning method that is well suited to small sample datasets and nonlinear response surfaces. Assume the existence of a potential function *f*(*x*) such that the function values *f* = [*f*(*x*_1_), *f*(*x*_2_), …, *f*(*x*_n_)] on any finite set *X* = [*x*_1_, *x*_2_, …, *x*_n_] follow a joint Gaussian distribution, as shown in Equation (3).

(3) $$p(f|X)=\chi (m(X),K(X,X^{\prime }))$$

where m(*X*) is the mean vector and *K*(*X*, *X*′) is the covariance matrix, the elements *K*_ij_ = *k*(*x*_i_, *x*_j_) of which are calculated using the kernel function.

The squared exponential kernel function is employed, as shown in Equation (4).

(4) $$k(x_{i},x_{j})=\sigma_{f}^{2}\exp(-\frac{1}{2l^{2}}{\left\|x_{i}-x_{j}\right\|}^{2})+\sigma_{n}^{2}\delta_{ij}$$

where *σ_f_*^2^ is the signal variance, *l* is the feature length scale, *σ_n_*^2^ is the noise variance, and *δ*_ij_ is the Kronecker delta function.

Given the observed data (*X*, *y*), for a new input x*, GPR uses Bayesian inference to obtain the predictive distribution, as shown in Equation (5).

(5) $$p(f*|x*,X,y)=\chi \left[\mu (x*),\sigma^{2}(x*)\right]$$

Bayesian optimization (BO) utilizes the aforementioned predictive distribution to guide the selection of the next trial point via a sampling function. The expected improvement (EI) is adopted as the sampling function, as shown in Equation (6).

(6) $$EI(x)=\left\{ \begin{array}{ll} \left[\mu_{best}-\mu \left(x\right)\right]\Phi (z)+\sigma (x)\phi (z) & \;\mathrm{if}\;\sigma (x)>0\\ 0 & \mathrm{if}\;\sigma (x)=0 \end{array}\right.$$

where $z=\left[\mu_{best}-\mu (x)\right]/\sigma (x)$, *Φ*(*z*) and *ϕ*(*z*) are the cumulative distribution function and probability density function of the standard normal distribution, respectively.

#### 2.3.4. Confirmatory Experiment of the Optimal Formulation

To independently validate the GPR-BO prediction, a confirmatory experiment was conducted using the optimal formulation. Three replicate specimens were freshly prepared following the same compaction procedure described in Section 2.2.1 and tested under the identical indoor lighting simulation conditions described in Section 2.2.2. The measured *T*_90_, *v*, and *η* values were compared against the GPR model predictions and their 95% confidence intervals.

## 3. RSM-Based Preliminary Screening of Candidate Materials

### 3.1. Thermal Conductivity Behavior

**Figure 7:**
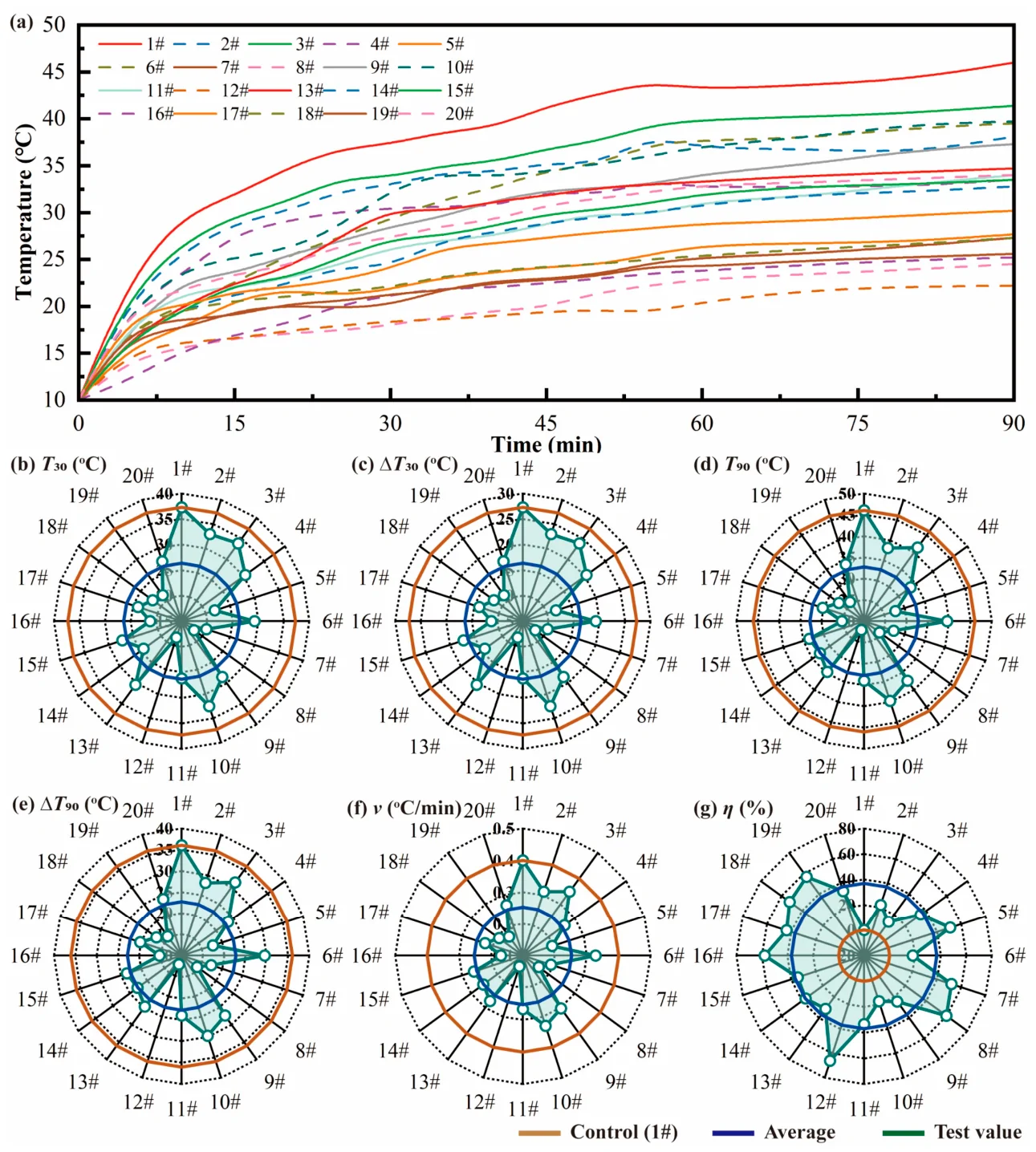
Test results and evaluation criteria for the surface temperature of the insulation layer (H = 0 cm): (**a**) Temperature rise curve; (**b**) *T*_30_; (**c**) ∆*T*_30_; (**d**) *T*_90_; (**e**) ∆*T*_90_; (**f**) *v*; (**g**) *η*.

**Figure 8:**
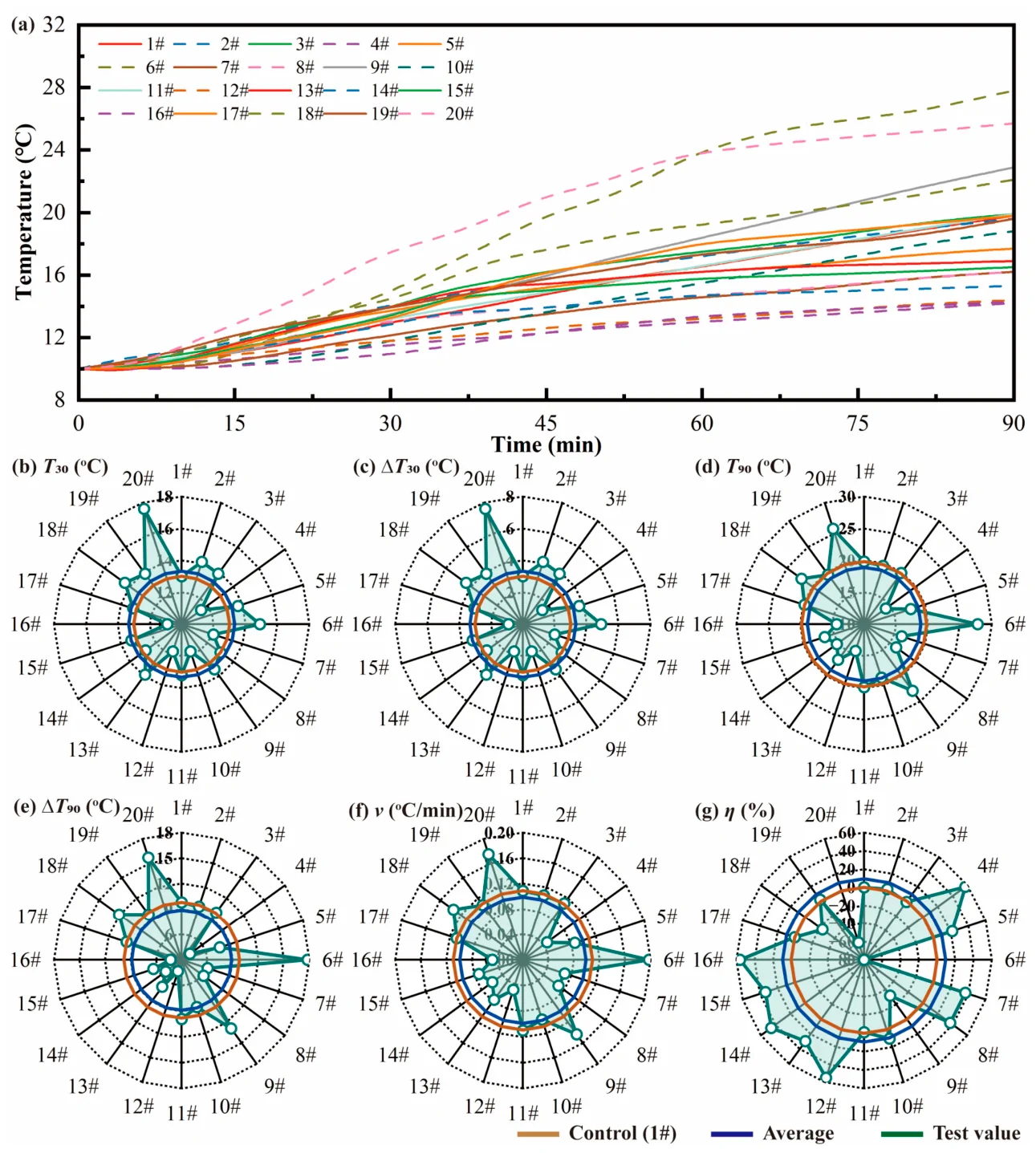
Test results and evaluation criteria for the bottom temperature of the insulation layer (H = 4 cm): (**a**) Temperature rise curve; (**b**) *T*_30_; (**c**) ∆*T*_30_; (**d**) *T*_90_; (**e**) ∆*T*_90_; (**f**) *v*; (**g**) *η*.

**Figure 9:**
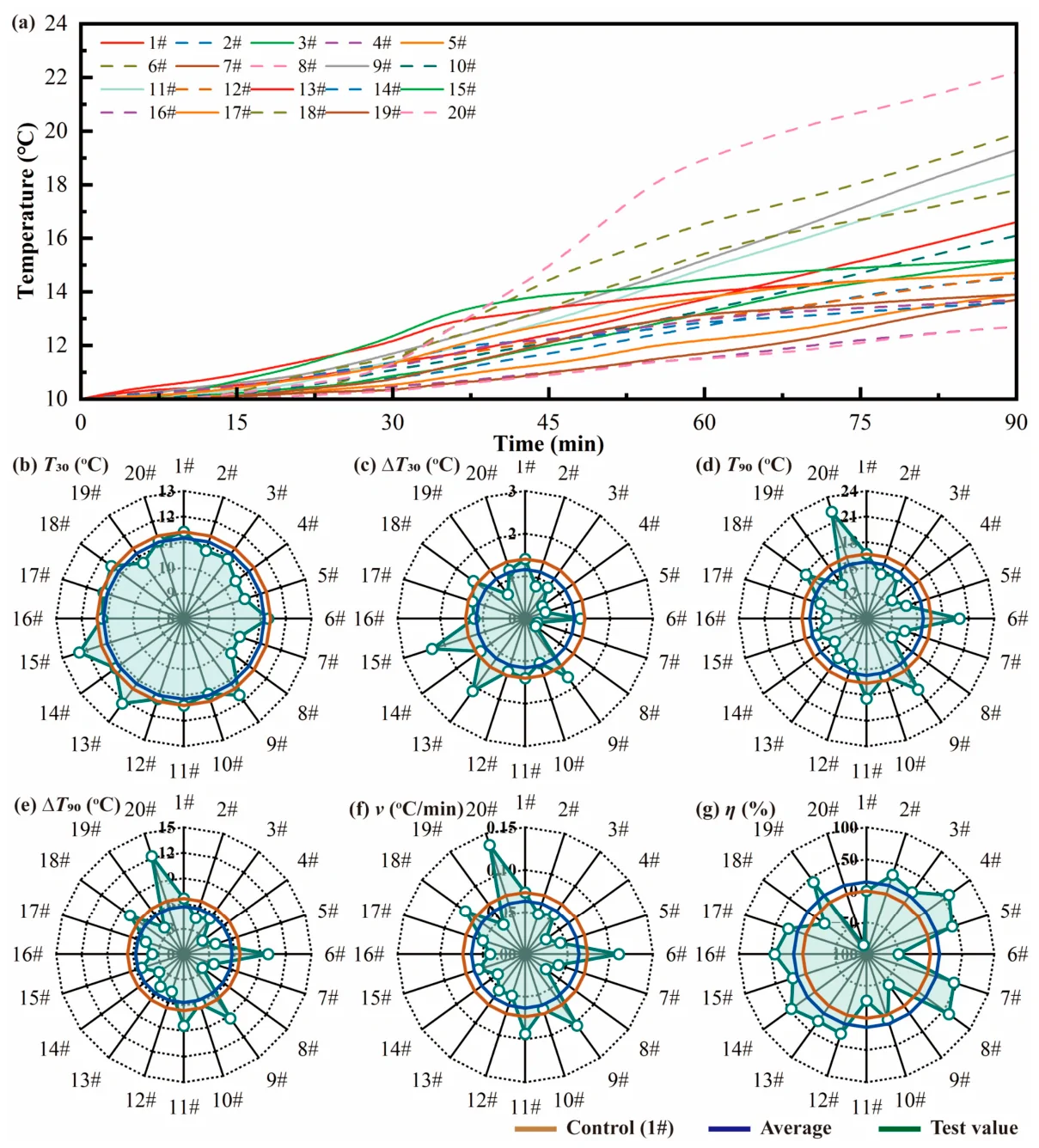
Test results and evaluation criteria for the bottom temperature of soil specimens (H = 10 cm): (**a**) Temperature rise curve; (**b**) *T*_30_; (**c**) ∆*T*_30_; (**d**) *T*_90_; (**e**) ∆*T*_90_; (**f**) *v*; (**g**) *η*.

The results of indoor lighting simulation tests conducted during the initial selection phase are shown in Figure 7, Figure 8 and Figure 9.

As shown in Figure 7, Figure 8 and Figure 9, all specimens exhibited a clear depth-dependent thermal response under continuous illumination. The temperature at 0 cm increased most rapidly, whereas the responses at 4 and 10 cm were progressively delayed, indicating that heat was gradually attenuated during downward transfer through the compacted insulation layer and the underlying soil. Under the present test configuration, the three monitoring positions represented the insulation-layer surface, the bottom of the insulation layer, and the bottom of the soil layer, respectively; all 20 groups were prepared and tested under the same preliminary screening framework.

The temperature evolution showed pronounced stratification. On average, the temperature at 0 cm increased from 10.0 °C to 26.3 °C after 30 min and further to 32.7 °C after 90 min; thus, 71.9% of the total 90 min temperature rise occurred within the first 30 min, indicating rapid surface heating. By contrast, the mean temperatures at 4 cm were 13.3 °C and 18.9 °C at 30 and 90 min, respectively, and those at 10 cm were only 11.2 °C and 15.7 °C. The thermal response in the lower layers was considerably slower: only 37.4% of the final temperature rise at 4 cm and 20.4% at 10 cm had developed by 30 min, demonstrating a pronounced lag effect in deep heat transfer. This confirms that the compacted multilayer structure not only reduced the magnitude of heating but also altered the temporal pathway of heat propagation.

Groups 4, 8, 12, and 16 all used hollow glass microspheres (O1) as the secondary skeleton. These groups consistently exhibited the lowest temperatures at all three monitoring depths. This behavior is consistent with the dual function of the thin-walled closed-cell structure of O1. The spherical glass shells can reflect part of the incident radiation, thereby reducing surface heat absorption. Meanwhile, the enclosed air cavities can act as discrete low-conductivity inclusions that extend conductive heat-transfer pathways. Similar mechanisms have been demonstrated for hollow glass microspheres in previous studies [15,16]. The O1-containing groups achieved the highest heat resistance efficiencies at both the surface and at depth. Specifically, Group 12 reached 66.1% at the surface, while Group 4 reached 57.1% at 4 cm and 59.1% at 10 cm. This pattern is consistent with the combined effects of radiative suppression and conductive-path interruption.

In contrast, Groups 6 and 20, which contained straw (B1) or wood chips (B2) in the secondary skeleton, showed strongly negative heat resistance efficiencies at depth. The elongated fibrous morphology of these materials provides a plausible explanation. Under compaction, fibers may preferentially align along the compaction axis and form low-resistance pathways parallel to the heat-flux direction. This behavior is consistent with the reported anisotropy in the thermal conductivity of compacted fibrous media [45]. The inferred thermal-bridging effect may be further enhanced by the low volumetric heat capacity of dry lignocellulosic biomass, as supported by the hygrothermal characterization of wood-fiber insulation [20].

Group 18, consisting of S1 + B2 + O2, illustrates a partial decoupling between surface optical suppression and internal conductive-path interruption. TiO_2_ (O2) was associated with moderate surface thermal suppression, with a surface *T*_90_ of 27.3 °C. However, B2 was associated with conductive pathways at depth, as indicated by *T*_90_ values of 22.1 °C at 4 cm and 17.8 °C at 10 cm. The surface optical suppression alone may be insufficient for effective through-thickness insulation if it is not accompanied by conductive-path interruption within the skeleton layer.

The secondary skeleton factor exerted the strongest control over thermal behavior: the span of its mean *T*_90_ values reached 11.18 °C at 0 cm, 9.13 °C at 4 cm, and 6.58 °C at 10 cm, all exceeding the corresponding spans of the primary skeleton and filling factors. The primary skeleton factor mainly governed the continuity of the internal conductive network; Groups 13–16 produced the lowest mean *T*_90_ values at both 4 and 10 cm, indicating that the load-bearing framework strongly affected downward heat propagation after compaction. The filling factor showed a more selective effect. Under the test conditions, the latent-heat mechanism of C1 was unlikely to be activated. Its modest positive contribution can be attributed to the suppression of convective heat transfer within inter-particle voids by liquid paraffin [42], together with sensible heat-capacity buffering.

Overall, the present results demonstrate that the thermal performance of the insulation layer was controlled by both surface heat accumulation and internal heat transmission, and neither aspect could be neglected. Groups 12, 16, 8, and 4 showed the most favorable balance between these two requirements and therefore emerged as the optimal candidates in the preliminary screening stage.

### 3.2. Factor Influence Analysis

**Figure 10:**
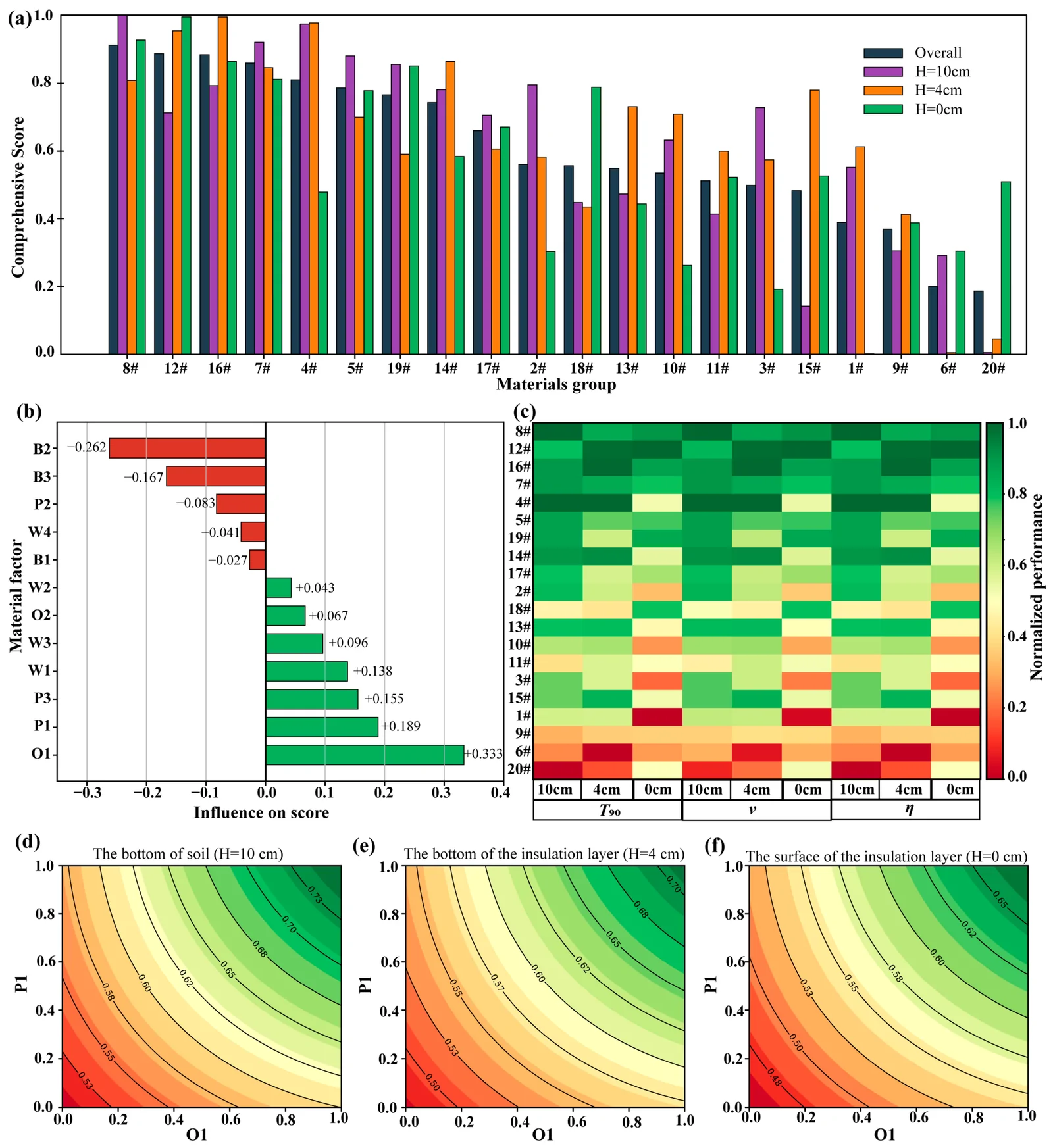
The factors influencing insulation materials: (**a**) Scores for different material combinations; (**b**) The influence of material factors; (**c**) The influence of material combinations on performance indicators; (**d**) The interaction effect between O1 and P1 (H = 10 cm); (**e**) The interaction effect between O1 and P1 (H = 4 cm); (**f**) The interaction effect between O1 and P1 (H = 0 cm).

The factors influencing insulation materials are shown in Figure 10.

The ranking of the 20 groups based on the comprehensive score is shown in Figure 10a. A clear gradient distribution can be observed: Groups 8, 12, 16, and 4 consistently exhibited the highest overall scores, whereas Groups 6 and 20 were located at the lower end of the ranking. This trend is consistent with the temperature-based evaluation discussed previously, confirming the reliability of the multi-index comprehensive assessment. The contribution of each material factor to the overall performance is illustrated in Figure 10b. Significant differences were observed among the factors.

Positive contributions were mainly associated with O1, P1, and P3, with influence coefficients of +0.333, +0.189, and +0.155, respectively. Among these, O1 showed the strongest positive effect. Welch’s *t*-test comparing groups with and without O1 confirmed that this effect was statistically significant (t = 6.226, *p* < 0.001, Cohen’s d = 1.862). The 95% confidence interval for the improvement in comprehensive score was +22.1% to +44.5%. This result remained significant after Bonferroni correction for all 13 material comparisons (*α*_adjusted_ = 0.0038). Negative contributions were mainly associated with B2, B3, and P2, with influence coefficients of −0.262, −0.167, and −0.083, respectively. None of the other individual material factors reached statistical significance at *α* = 0.05. The full statistical results are provided in Table 3. Overall, the secondary skeleton factor exhibited the largest variation range, indicating that it was the most sensitive factor controlling the thermal response.

The normalized performance distribution of all groups across different depths and indices is presented in Figure 10c. A consistent pattern can be identified: high-performing groups maintained relatively high normalized values across *T*_90_, heating rate *v*, and heat resistance efficiency *η* at all three depths, whereas low-performing groups showed significant fluctuations, particularly at 4 cm and 10 cm, where the normalized values decreased sharply. This indicates that poor-performing combinations failed to effectively suppress internal heat transfer. It is also notable that some groups exhibited relatively high normalized values at 0 cm but much lower values at deeper positions, suggesting that surface cooling alone could not ensure overall insulation effectiveness.

The response surface analysis further reveals the interaction between key factors (Figure 10d–f). The combined effects of the two dominant variables exhibit a clear nonlinear trend. At all three depths, comprehensive performance increased gradually with the simultaneous increase of both variables; the smooth, convex contour lines indicate a synergistic interaction rather than a simple additive effect. The improvement was more pronounced in the upper region of the response surface, where both variables approached higher levels, suggesting that the formation of an effective thermal barrier required the coordinated contribution of both factors. The sensitivity of the response surface decreased with depth: at 0 cm, the contour gradient was steeper, indicating greater sensitivity to factor variation; at 4 cm, the gradient became moderate; at 10 cm, the surface became smoother, suggesting that the influence of individual factors was partially attenuated during downward heat propagation. This depth-dependent attenuation reflects the cumulative and dissipative nature of heat conduction within the multilayer structure.

For each material factor, the comprehensive scores were compared between specimens with the material present and absent. The influence magnitude was calculated as the difference in mean comprehensive scores, as shown in Table 3.

### 3.3. Material Screening and Mechanism Interpretation

Based on the comprehensive RSM analysis, the top-performing material combinations were identified. Table 4 summarizes the screening results showing the top 10 material combinations ranked by overall comprehensive score.

The screening results in Table 4 indicate a consistent material-design logic based on the three heat-transfer mechanisms. Expanded clay aggregates (P1) were selected as the primary skeleton. Their high porosity, greater than 60%, and granular morphology provide a continuous low-conductivity framework. This framework can resist pore collapse under compaction and therefore provides the structural basis for the secondary skeleton and fillers to function effectively. Hollow glass microspheres (O1) were selected as the secondary skeleton. This selection was supported by their dual reflectance–conductance mechanism. O1 showed the strongest and statistically significant individual contribution to overall performance, with an increase of 33.31%. Fly ash (W1) was selected as the filling material. Its fine spherical particles can fill interstitial spaces without forming continuous thermal bridges. This helps modestly reduce effective conductivity while preserving the insulating pore structure.

The multi-depth temperature data and factor-influence analysis suggest that thermal regulation arises from three complementary processes. Surface heat input suppression is most plausibly associated with optical components such as hollow glass microspheres and titanium dioxide, whose radiative-barrier and infrared-reflective performance has been independently demonstrated [15,16,18]. Bulk heat-transfer path interruption is most plausibly associated with porous lightweight skeletons, where the reduction in effective thermal conductivity can be attributed to pore-mediated extension of conductive pathways [13,14,46]. Thermal buffering is most plausibly associated with fine fillers contributing to heat-capacity effects and, potentially, latent-heat storage [26,27]. It should be noted that evidence for these mechanisms is indirect, based on spatial and temporal temperature patterns at three depths rather than direct measurements of reflectance, emissivity, specific heat capacity, or heat flux. Nevertheless, the groups with the highest comprehensive scores consistently incorporated materials from multiple functional categories, supporting the multi-mechanism interpretation.

## 4. GPR-BO-Based Mixture Optimization

### 4.1. Construction and Analysis of GPR Models

**Figure 11:**
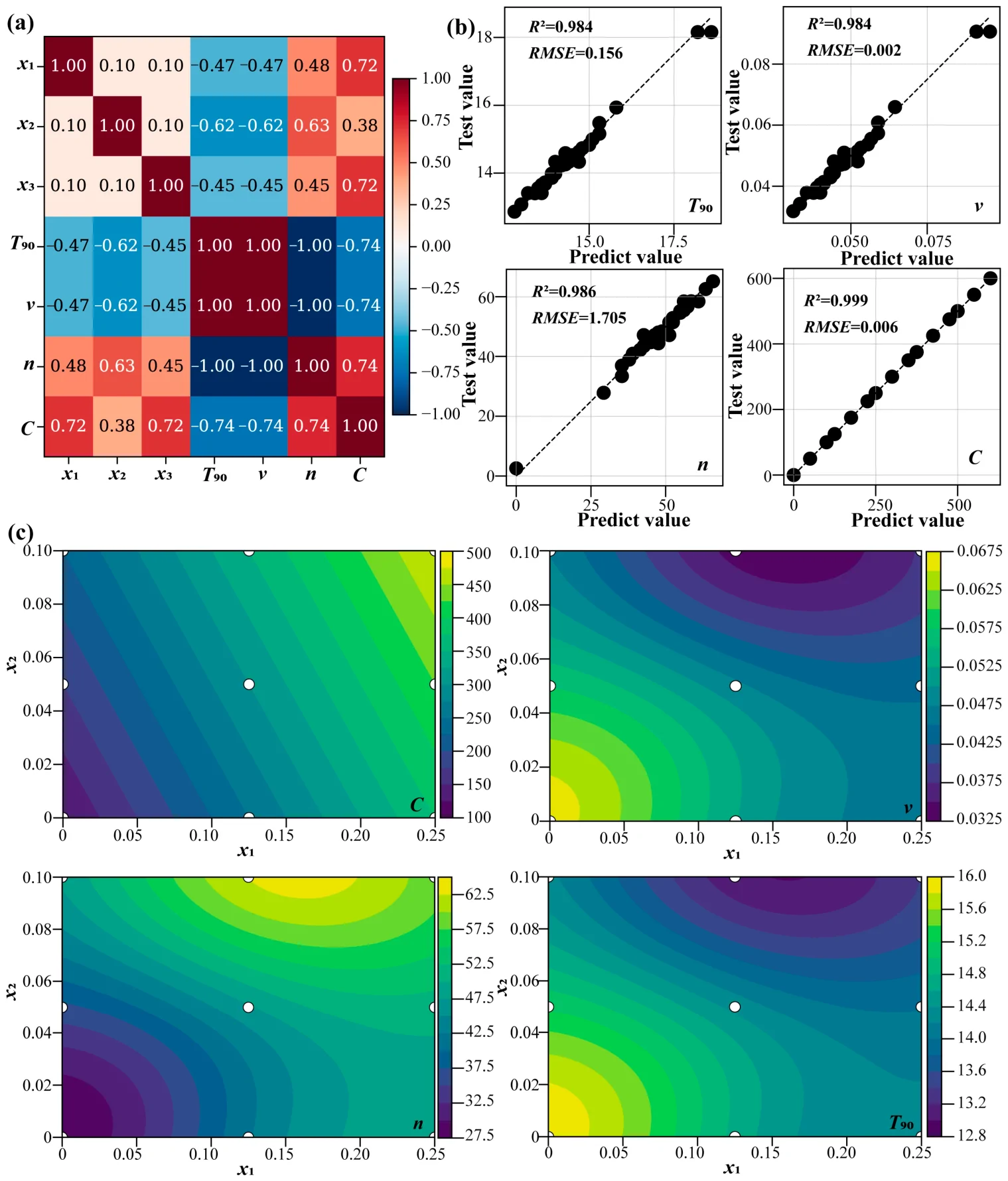
Prediction results of the GPR model: (**a**) Heatmap of variable correlations; (**b**) Comparison of predicted values with test values; (**c**) Response surface analysis (*x*_3_ = 0.125).

A GPR model was developed based on the 32 initial experimental runs. The relationships among variables are presented in Figure 11. As shown in Figure 11a, components *x*_1_ and *x*_3_ have a dominant influence on the output variables *η* and *C*, implying that *C* can be accurately predicted from the formulation parameters. *T*_90_ and v exhibit an extremely high correlation (*r* = 0.99), suggesting that these two response variables essentially reflect the same underlying phenomenon. Component *x*_1_ is negatively correlated with *T*_90_ and *v*; as *x*_1_ increases from 0 to 0.25, *T*_90_ decreases by approximately 3–4 °C.

As shown in Figure 11b, the plot of predicted versus actual values shows that all data points are closely clustered around the ideal diagonal line (*y* = *x*), indicating excellent model fit. The prediction accuracy for *T*_90_, *v*, and *η* on the training set was high. The corresponding *R*^2^ values were 0.984, 0.984, and 0.986, respectively, and the RMSE values were 0.156, 0.002, and 1.705, respectively. For *T*_90_ and *v*, the optimized kernel hyperparameters were *σ*_f_^2^ = 0.85^2^, RBF length scale *l* = 1.01, and white-noise term *σ*_n_ = 0.057. For *η*, the corresponding values were *σ*_f_^2^ = 0.85^2^, *l* = 1.01, and *σ*_n_ = 0.051. The similar length scales across the three responses indicate that these outputs respond to formulation changes with comparable smoothness. This is consistent with their shared physical origin. For *C*, the model achieved an *R*^2^ of 1.000 and an RMSE of 0.006. These results confirm the deterministic relationship between *C* and the formulation components. The optimized hyperparameters were *σ*_f_^2^ = 28.1^2^, *l* = 1.14, and *σ*_n_ ≈ 0.

The response surface (Figure 11c) illustrates the variation in the output variables in the input space, focusing on the *x*_1_–*x*_2_ plane at a fixed value of *x*_3_. The response surfaces for *T*_90_ and *v* exhibit similar patterns: both response variables decrease monotonically as *x*_1_ and *x*_2_ increase, with the minimum at the corner where both reach their maximum levels (*x*_1_ = 0.25, *x*_2_ = 0.10) and the maximum at the origin (*x*_1_ = *x*_2_ = 0). Prediction uncertainty is relatively low near the training data points but increases significantly in regions far from the experimental observations, which is a typical characteristic of Gaussian processes and highlights the importance of adaptive sampling. The response surface for *η* exhibits more complex behavior compared to those for *T*_90_ and *v*, with a non-monotonic relationship and local minima and maxima occurring within the design space. The range of *η* extends from 0 to 66, with the minimum at the origin and the maximum at (*x*_1_ = 0.25, *x*_2_ = 0.10, *x*_3_ = 0.25). The response surface for *C* exhibits an approximately linear gradient ranging from 0 to 600 in the input space, implying that *C* can be precisely predicted from the formulation without accounting for modeling uncertainty. The uncertainty surface reveals that prediction uncertainty is lowest near existing data points and gradually increases along the boundaries of the design space; this pattern is particularly pronounced for *η*, whose response surface exhibits the most complex curvature.

The monotonic decrease in *T*_90_ and *v* with increasing *x*_1_ and *x*_2_ is physically consistent with the RSM screening results. A higher proportion of the porous primary skeleton lengthens conductive heat-transfer pathways. Similarly, a higher proportion of the optical secondary skeleton suppresses radiative absorption. Both effects reduce the net heat flux reaching the measurement positions. In contrast, *η* showed a more complex and non-monotonic response. This behavior reflects the multi-dimensional nature of the desirability index, which integrates thermal performance with additional constraints. The curvature of *η* also captures the trade-off structure identified in the correlation analysis.

### 4.2. Material Optimization Based on GPR-BO

**Figure 12:**
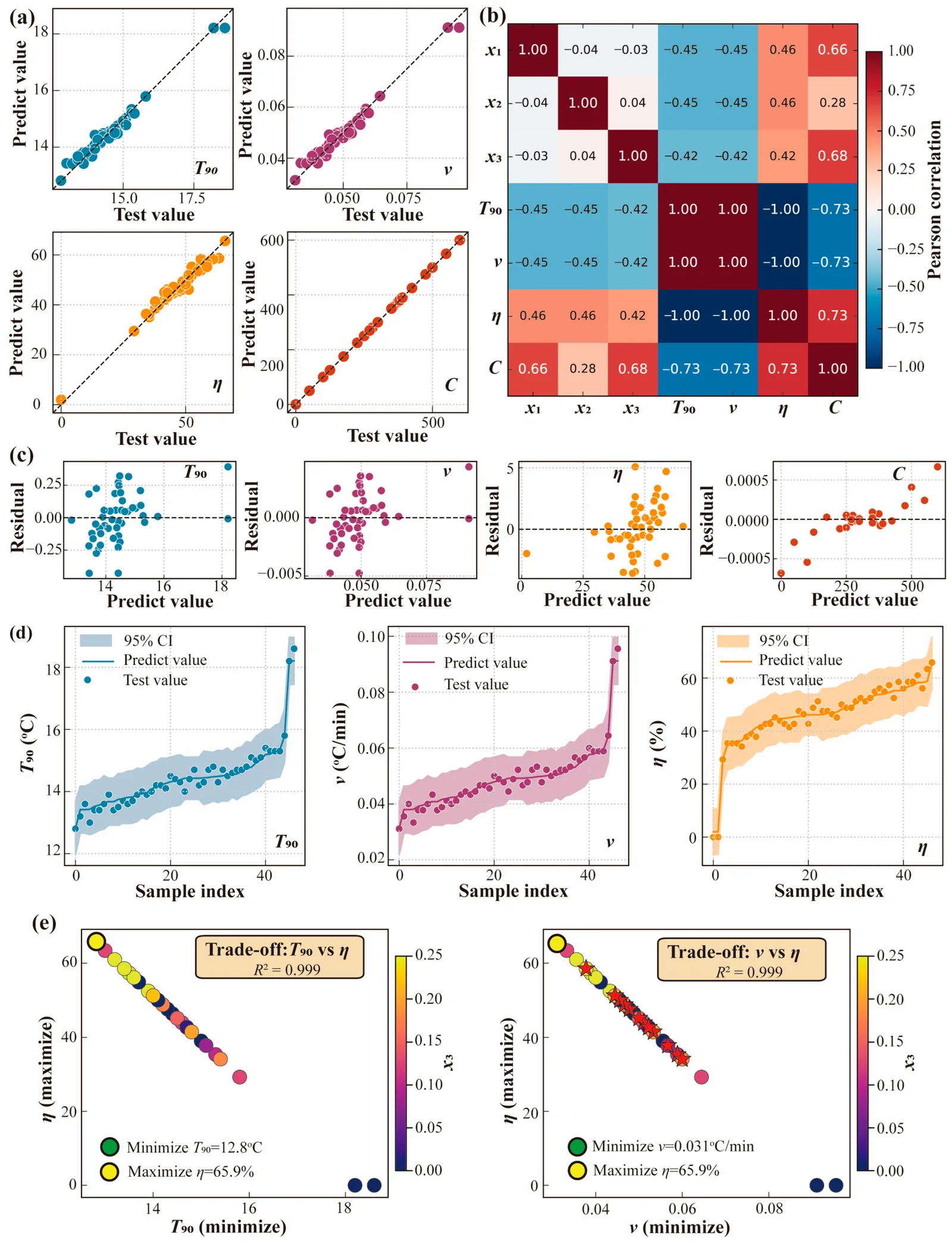
Prediction results of the GPR-BO model: (**a**) Comparison of predicted and test values; (**b**) Correlation heatmap of input variables and output variables; (**c**) Residual analysis; (**d**) Prediction uncertainty with 95% confidence intervals; (**e**) Pareto trade-off analysis, red stars—BO experiments.

After constructing the GPR model, Bayesian optimization was performed using the Expected Improvement (EI) acquisition function to identify promising candidates. Following incorporation of the results from BO-01 to BO-15, the GPR model was updated. The newly sampled points exhibited clustering behavior in the high-microsphere region, indicating that the model had identified a promising optimal region. The prediction results of the GPR-BO model are shown in Figure 12.

As shown in Figure 12a, the predicted and actual values for all output variables are tightly aligned along the ideal prediction line, demonstrating the predictive performance of the GPR model after incorporating all 47 samples (32 initial + 15 BO iterations). After incorporating the 15 BO iterations, the GPR model was retrained using the full 47-sample dataset. The retrained model achieved R^2^ values of 0.971 for *T*_90_ and *v*, 0.999 for *η*, and 1.000 for *C*. The small changes relative to the 32-sample model indicate that the initial surrogate captured the dominant response trends.

The predictions for *T*_90_ and *v* exhibit near-perfect agreement with the experimental values, with coefficients of determination exceeding 0.95, indicating that the model effectively captures their dependence on formulation parameters. For *η* and *C*, despite their broader numerical ranges, the model maintains robust and unbiased predictive performance across the entire domain.

The correlation heatmap in Figure 12b further elucidates the relationships between input and output variables. *T*_90_ and *v* display an almost perfect positive correlation, suggesting that they represent closely related response characteristics. In addition, *x*_3_ shows a strong positive correlation with *η*, indicating that this parameter plays a dominant role in governing system performance. A sensitivity ranking based on the magnitudes of Pearson correlation coefficients shows that *x*_3_ had the strongest influence on *η*, with |*r*| = 0.93. This was followed by the effects of *x*_1_ on *T*_90_ and *v*, with |*r*| = 0.58. The effect of *x*_2_ was secondary, with |*r*| ranging from 0.18 to 0.55. This ranking is consistent with the factor-influence analysis from the RSM screening stage. It further indicates that the volumetric proportion of the filling component is the most sensitive factor for tuning overall performance.

Meanwhile, *T*_90_, *v*, and *η* exhibit a pronounced negative correlation, highlighting inherent trade-offs among these responses. As illustrated in Figure 12c, the residuals are randomly distributed with no discernible structural pattern, confirming that the assumption of homoscedasticity is satisfied. Figure 12d further demonstrates a key advantage of the GPR model: its ability to provide both predictions and associated uncertainty estimates. In regions with dense training data, the confidence intervals are narrow, reflecting high reliability, whereas in sparse or boundary regions they expand significantly, consistent with the limited extrapolation capability of surrogate models. For the optimal formulation, with *x*_1_ = 0.125, *x*_2_ = 0.100, and *x*_3_= 0.250, the GPR model predicted *T*_90_ = 12.80 °C, with a 95% confidence interval of approximately 12.44–13.16 °C. The predicted value of *v* was 0.0311 °C/min, with a 95% confidence interval of 0.027–0.035 °C/min. The predicted value of *η* was 65.85%, with a 95% confidence interval of 63.70–68.00%. These intervals were derived from the posterior predictive variance of the GPR model. They quantify the remaining model uncertainty at the recommended operating point and provide realistic bounds for the expected experimental outcomes.

The distribution shown in Figure 12e highlights the critical role of *x*_3_ in regulating trade-offs among the responses. Lower *x*_3_ content corresponds to lower values of *T*_90_, *v*, and *η*, whereas increasing *x*_3_ results in simultaneous increases in these variables, confirming that *x*_3_ is a key control parameter governing the balance between performance metrics. In practical applications requiring higher *η* values, increasing *x*_3_ is necessary, although this leads to higher *T*_90_ and *v*.

Based on the Pareto front analysis, the optimal formulation is identified as *x*_1_ = 0.125, *x*_2_ = 0.100, and *x*_3_ = 0.250. The strong positive relationships between *T*_90_ and *v*, as well as between *x*_3_ and *η*, enable coordinated optimization through adjustment of *x*_3_. As indicated in Figure 12e, this formulation lies near an inflection region of the Pareto front, representing an optimal balance among competing objectives. It maintains a low *T*_90_ while achieving a high *η*, making it the most favorable overall solution. Additional candidate formulations are provided in Table 5.

Unit prices for cost calculation were obtained from Alibaba 1688 and Baidu Ai Procurement (2026 Q2): P1 = 1000 yuan/t, O1 = 300 yuan/t, W1 = 80 yuan/t. C = Σ (unit price × dosage). Soil (S1/S2) assumed zero material cost on-site.

Independent validation tests showed that the measured values of all three response variables fell within the 95% confidence intervals predicted by the GPR model. The GPR-predicted values were *T*_90_ = 12.80 °C, *v* = 0.0311 °C/min, and *η* = 65.85%. The measured values were *T*_90_ = 12.86 ± 0.17 °C, *v* = 0.032 ± 0.0002 °C/min, and *η* = 65.08 ± 2.13%. The deviations between measured and predicted values were small: Δ*T*_90_ = +0.06 °C, Δ*v* = +0.0009 °C/min, and Δ*η* = −0.77%. These results confirm that the GPR-BO surrogate model provides reliable predictions for the optimal formulation and that the formulation obtained from the two-stage optimization framework is experimentally reproducible.

## 5. Conclusions

A two-stage framework integrating RSM screening with GPR-BO was established for the systematic design of composite thermal insulation layers for mine ecological restoration under simulated solar radiation. Fifteen candidate materials across six categories were evaluated, and an optimal multi-component formulation was identified. The main conclusions are as follows.

(1) Among the six material categories, hollow glass microspheres (O1) emerged as the dominant thermal-regulation component, improving comprehensive thermal performance by +33.31%. This effect was the only one confirmed as statistically significant through formal hypothesis testing and remained significant after Bonferroni correction for 13 comparisons, identifying O1 as a key functional component for compacted mine restoration insulation layers.
(2) The proposed RSM–GPR-BO framework was effective for formulation design under limited experimental resources. The RSM stage identified the dominant factor and provided directional trends for other candidates. The subsequent GPR-BO stage refined the mixture proportions, yielding an optimal formulation of *x*_1_ = 0.125, *x*_2_ = 0.100, and *x*_3_ = 0.250. The GPR model predicted *T*_90_ = 12.80 °C, with a 95% confidence interval of approximately 12.44–13.16 °C. The predicted value of *v* was 0.0311 °C/min, with a 95% confidence interval of 0.027–0.035 °C/min. The predicted value of *η* was 65.85%, with a 95% confidence interval of 63.70–68.00%. The sequential optimization process mitigated the limitations of the initial small-sample surrogate model through iterative data acquisition and model updating.
(3) The optimal formulation integrates three functional roles: a porous primary skeleton (P1) for conductive-path interruption, an optical secondary skeleton (O1) for radiative suppression, and a low-cost industrial waste filler (W1, fly ash) for interstitial filling. The estimated material cost is 475 yuan/t based on 2026 Q2 market prices from Alibaba 1688 and Baidu Ai Procurement. This multi-mechanism architecture outperforms single-category formulations, supporting the proposed synergy hypothesis.
(4) Several limitations should be acknowledged. The mechanistic interpretation of thermal regulation is based on multi-depth temperature responses rather than direct measurements of radiative, thermal, or transport properties. An independent confirmatory experiment using three freshly prepared replicate specimens verified the optimal formulation: the measured performance (*T*_90_ = 12.86 ± 0.17 °C, *v* = 0.0318 ± 0.0019 °C/min, *η* = 65.08 ± 2.13%) was within the GPR 95% confidence intervals for all three response variables, confirming the reliability of the model prediction. In addition, all experiments were conducted under controlled laboratory conditions with fixed irradiance (200 W/m^2^).

Future work should address five aspects: (i) field validation under natural solar radiation cycles with vegetation establishment monitoring over at least one full annual cycle; (ii) wetting–drying and freeze–thaw durability testing; (iii) direct characterization of reflectance, emissivity, heat capacity, and heat flux to quantify individual mechanism contributions; (iv) leaching toxicity testing of the fly ash component; and (v) vegetation establishment trials combining the optimal insulation layer formulation with native plant species.

## Figures and Tables

**Table 1:** The technical characteristics of thermal insulation materials.

Material Types	Materials	Technical Characteristics
Solid waste materials	Fly ash	Density: 2.48 g/cm^3^; Thermal conductivity: 0.30 W/(m·K)
	Phosphogypsum	Density: 2.46 g/cm^3^; Thermal conductivity: 0.30 W/(m·K)
	Coal slag	Density: 2.11 g/cm^3^; Thermal conductivity: 0.31 W/(m·K); Porosity: 56%
	Coal gangue	Density: 2.30 g/cm^3^; Thermal conductivity: 0.27 W/(m·K); Porosity: 45%
Biomass materials	Straw	Bulk density: 0.12 g/cm^3^; Thermal conductivity: 0.09 W/(m·K)
	Wood chips	Bulk density: 0.28 g/cm^3^; Thermal conductivity: 0.21 W/(m·K)
	Recycled wood flour	Bulk density: 0.33 g/cm^3^; Thermal conductivity: 0.21 W/(m·K)
Porous materials	Expanded clay aggregates	Bulk density: 0.80 g/cm^3^; Thermal conductivity: 0.2 W/(m·K); Porosity: >60%
	Diatomaceous earth	Bulk density: 0.55 g/cm^3^; Thermal conductivity: 0.015 W/(m·K); Porosity: >80%
	Aerogel	Bulk density: 0.10 g/cm^3^; Thermal conductivity: 0.021 W/(m·K); Porosity: >90%
Optical materials	Hollow glass microspheres	Density: 0.41 g/cm^3^; Thermal conductivity: 0.06 W/(m·K); Refractive index: 1.54
	Titanium dioxide	TiO_2_ content: ≥98.9%; Density: 4.05 g/cm^3^; Average particle size: 0.20–0.26 μm; Whiteness: ≥99%; TCS value: ≥1900
Phase-change materials	Phase change paraffin	Phase transition temperature: 5 °C; Enthalpy: 220 J/kg; Density: 0.8 g/cm^3^; Thermal conductivity: 0.2 W/(m·K); Specific heat capacity: 2.0 J/(kg·K)

**Table 2:** Preliminary selection comprising 20 groups of materials.

Group Number	Skeleton Material	Secondary Skeleton Material	Filling Material
1	S1	S2	S2
2	S1	S2 + B1	S2 + W1
3	S1	S2 + W2	S2 + P2
4	S1	S2 + O1	S2 + C1
5	S1 + P1	S2	S2 + W1
6	S1 + P1	S2 + B1	S2 + P2
7	S1 + P1	S2 + W2	S2 + C1
8	S1 + P1	S2 + O1	S2
9	S1 + W4	S2	S2 + P2
10	S1 + W4	S2 + B1	S2 + C1
11	S1 + W4	S2 + W2	S2
12	S1 + W4	S2 + O1	S2 + W1
13	S1 + W3	S2	S2 + C1
14	S1 + W3	S2 + B1	S2
15	S1 + W3	S2 + W2	S2 + W1
16	S1 + W3	S2 + O1	S2 + P2
17	S1	S2 + P3	S2 + B3
18	S1	S2 + B2	S2 + O2
19	S1 + P1	S2 + P3	S2 + O2
20	S1 + W4	S2 + B2	S2 + B3

**Table 3:** Statistical analysis of material effects.

Material Factor	Score with Factor	Score Without Factor	Influence (%)	*p*	Cohen’s d
O1	0.87	0.54	+33.31	<0.001	1.862
P1	0.70	0.57	+13.01	0.388	0.592
C1	0.69	0.59	+10.12	0.364	0.453
P3	0.71	0.60	+11.77	0.194	0.525
W3	0.65	0.57	+8.00	0.534	0.319
W1	0.68	0.59	+9.00	0.451	0.401
B2	0.37	0.63	−26.23	0.380	<0.2
B3	0.42	0.63	−20.45	0.545	<0.2
P2	0.49	0.64	−14.95	0.390	<0.2
W4	0.50	0.64	−14.64	0.295	<0.2

**Table 4:** Preliminary ranking of candidate materials based on the comprehensive thermal insulation score.

Rank	Groups	Primary Skeleton	Secondary Skeleton	Filling	Overall Score	Best Depth
1	8#	S1 + P1	S2 + O1	S2	0.9125	10 cm (1.0010)
2	12#	S1 + W4	S2 + O1	S2 + W1	0.8877	0 cm (0.9958)
3	16#	S1 + W3	S2 + O1	S2 + P2	0.8845	4 cm (0.9955)
4	7#	S1 + P1	S2 + W2	S2 + C1	0.8595	10 cm (0.9210)
5	4#	S1	S2 + O1	S2 + C1	0.8103	4 cm (0.9778)
6	5#	S1 + P1	S2	S2 + W1	0.7864	10 cm (0.8812)
7	19#	S1 + P1	S2 + P3	S2 + O2	0.7658	10 cm (0.8557)
8	14#	S1 + W3	S2 + B1	S2	0.7435	4 cm (0.8646)
9	17#	S1	S2 + P3	S2 + B3	0.6607	0 cm (0.6711)
10	2#	S1	S2 + B1	S2 + W1	0.5606	10 cm (0.7960)

**Table 5:** Comparison of alternative material combinations.

Priority	*x*_1_	*x*_2_	*x*_3_	*T*_90_ (°C)	*v* (°C/Min)	*n* (%)	*C* (Yuan/t)
1st	0.125	0.100	0.250	12.8	0.031	65.85	475
2nd	0.125	0.100	0.125	13.0	0.033	63.41	350
3rd	0.250	0.100	0.125	13.4	0.038	58.54	475
4th	0.250	0.000	0.250	13.4	0.038	58.54	500
5th	0.125	0.100	0.000	13.6	0.040	56.10	225
6th	0.000	0.100	0.000	13.9	0.043	52.44	100

## Data Availability

The original contributions presented in this study are included in the article. Further inquiries can be directed to the corresponding authors.
